# Artificial intelligence-enabled retinal vasculometry for prediction of circulatory mortality, myocardial infarction and stroke

**DOI:** 10.1136/bjo-2022-321842

**Published:** 2022-08-24

**Authors:** Alicja Regina Rudnicka, Roshan Welikala, Sarah Barman, Paul J Foster, Robert Luben, Shabina Hayat, Kay-Tee Khaw, Peter Whincup, David Strachan, Christopher G Owen

**Affiliations:** 1 Population Health Research Institute, St George's University of London, London, UK; 2 Faculty of Science, Engineering and Computing, Kingston University, Kingston-Upon-Thames, UK; 3 NIHR Biomedical Research Centre at Moorfields Eye Hospital and UCL Institute of Ophthalmology, University College London, London, UK; 4 MRC Epidemiology Unit, Cambridge University, Cambridge, UK; 5 Department of Psychiatry, Cambridge Public Health, University of Cambridge School of Clinical Medicine, Cambridge, UK

**Keywords:** Imaging, Retina, Diagnostic tests/Investigation, Epidemiology, Public health

## Abstract

**Aims:**

We examine whether inclusion of artificial intelligence (AI)-enabled retinal vasculometry (RV) improves existing risk algorithms for incident stroke, myocardial infarction (MI) and circulatory mortality.

**Methods:**

AI-enabled retinal vessel image analysis processed images from 88 052 UK Biobank (UKB) participants (aged 40–69 years at image capture) and 7411 European Prospective Investigation into Cancer (EPIC)-Norfolk participants (aged 48–92). Retinal arteriolar and venular width, tortuosity and area were extracted. Prediction models were developed in UKB using multivariable Cox proportional hazards regression for circulatory mortality, incident stroke and MI, and externally validated in EPIC-Norfolk. Model performance was assessed using optimism adjusted calibration, C-statistics and R^2^ statistics. Performance of Framingham risk scores (FRS) for incident stroke and incident MI, with addition of RV to FRS, were compared with a simpler model based on RV, age, smoking status and medical history (antihypertensive/cholesterol lowering medication, diabetes, prevalent stroke/MI).

**Results:**

UKB prognostic models were developed on 65 144 participants (mean age 56.8; median follow-up 7.7 years) and validated in 5862 EPIC-Norfolk participants (67.6, 9.1 years, respectively). Prediction models for circulatory mortality in men and women had optimism adjusted C-statistics and R^2^ statistics between 0.75–0.77 and 0.33–0.44, respectively. For incident stroke and MI, addition of RV to FRS did not improve model performance in either cohort. However, the simpler RV model performed equally or better than FRS.

**Conclusion:**

RV offers an alternative predictive biomarker to traditional risk-scores for vascular health, without the need for blood sampling or blood pressure measurement. Further work is needed to examine RV in population screening to triage individuals at high-risk.

**WHAT IS ALREADY KNOWN ON THIS TOPIC**

- Population screening for myocardial infarction (MI) and stroke using risk prediction tools exist but have limited uptake; risk scores for circulator mortality do not exist.

**WHAT THIS STUDY ADDS**

- Risk models developed in UK Biobank (validated in European Prospective Investigation into Cancer-Norfolk) using artificial intelligence (AI)-enabled retinal vasculometry (RV), age, history of cardiovascular disease, use of hypertensive medication and smoking yielded high predictive test performance for circulatory mortality.
- Risk scores for MI and stroke performed similarly to established risk scores.

**HOW THIS STUDY MIGHT AFFECT RESEARCH, PRACTICE OR POLICY**

- AI-enabled RV extraction offers a non-invasive prognostic biomarker of vascular health that does not require blood sampling or blood pressure measurement, and potentially has greater community reach to identify individuals at medium-high risk requiring further clinical assessment.

## Introduction

Circulatory mortality, including cardiovascular disease (CVD), coronary heart disease (CHD), heart failure and stroke, is a major cause of morbidity and mortality worldwide.^1 2^ A large number of risk algorithms exist to predict CVD,^3^ and the addition of fixed and modifiable risk factor phenotypes have been evaluated, but have so far shown little improvement in CVD prediction.^4–6^ Machine learning techniques incorporating 473 potential risk factors for the prediction CVD in the UK Biobank (UKB) cohort yielded areas-under-the-curve (AUC) from receiver operating characteristic curve of 0.774, compared with AUC of 0.724 for Framingham risk scores (FRS).^7^ Other CVD risk scores, using different CVD outcome definitions, have already been evaluated in UKB including the European Systemic Coronary Risk Evaluation (SCORE),^8^ QRISK3^9^ and American College of Cardiology/American Heart Association^10^ risk score with C-statistic values of 0.775, 0.739 and 0.736, respectively.^6^

Examination of retinal blood vessels (arterioles and venules) may offer a microvascular phenotype more indicative of the presence of early circulatory related disease processes, providing a non-invasive window on the circulatory system. Narrow retinal arterioles show a clear association with higher blood pressure (BP), hypertension and with incident CVD.^10^ Arteriolar vessel width narrowing and venular widening may be important for mortality, stroke^10^ and CHD incidence,^11^ but there are inconsistencies in the literature,^12 13^ such as retinal vessel associations with CVD risk in women but not in men.^10 14^ Other features of retinal vasculometry (RV), such as vessel tortuosity, may offer more discerning markers of vascular status but remain little studied at scale.^15 16^ Unfortunately, machine learning approaches do not currently clarify which features of RV are important, although they may do in the future.

We developed a fully automated artificial intelligence(AI)-enabled system (QUantitative Analysis of Retinal vessels Topology and siZe (QUARTZ)) for examining the retinal vascular tree, which overcomes many of the difficulties of earlier approaches, allowing detailed vasculometry quantification in large population studies.^17–19^ In the subset of UKB who underwent retinal imaging,^20^ and in the European Prospective Investigation into Cancer (EPIC)-Norfolk^15^ cohorts, we examine detailed characterisation of RV as a non-invasive maker of vascular health in relation to circulatory mortality prediction. In addition, we provide findings for FRS for stroke,^21^ and myocardial infarction (MI)^22^ in the same subset that underwent retinal imaging, and assess the incremental value of adding RV to FRS for incident stroke and MI.

## Materials and methods

*UKB* is a prospective cohort study for which baseline biomedical and physical assessments were carried out 2006–2013, in 502 682 adults aged 40–69 years recruited from 22 UK centres.^23^ Ocular assessments occurred during the latter phase (2009–2013; seven centres) and included visual acuity, autorefraction, digital fundus photography with the Topcon 3D-OCT 1000 Mark 2.^20^ Non-mydriatic 45° digital colour images, centred on the fovea were available for 88 052 participants.

*EPIC-Norfolk* was the UK component of the European Prospective Investigation into Cancer (EPIC) study.^24 25^ Here, we focus on data from the third clinical follow-up (2004–2011)^25^ on 8603 participants aged 48–92 years who underwent a biomedical and eye examination similar to that of UKB (online supplemental material for further details).^15^

10.1136/bjo-2022-321842.supp1 Supplementary data

### Health outcomes

The primary outcome was circulatory mortality as defined using International Classification of Diseases (ICD) (ICD-10 codes I00-I99 and ICD-9 390-459) coded death registry data from the Office for National Statistics and the Health and Social Care Information Centre (now NHS Digital) for England and Wales, and the Information Services Department for Scotland, provided information on date and cause(s) of death to 31 January 2018 for UKB and 31 March 2018 for EPIC-Norfolk. Incident MI and stroke events after retinal image capture were based on medical records linkage with hospital diagnoses of non-fatal events, supplemented with participant health and lifestyle questionnaire data from repeat surveys in UKB and EPIC-Norfolk (2012–2018). ICD-10 codes I21-I25 (or ICD-9 codes 410, 411, 412 429.79) were used for fatal and non-fatal MI; and ICD-10 codes I60, 61, 63, 64 (or ICD-9 codes 430, 431, 434, 436) for ischaemic and haemorrhagic stroke (see Algorithmically defined health outcomes at https://www.ukbiobank.ac.uk/enable-your-research/about-our-data/health-related-outcomes-data).

### AI-enabled retinal image processing

A validated, fully automated AI-enabled system (QUARTZ)^17–19^ extracted thousands of measures of retinal vessel width, tortuosity and area from the whole retinal image. Supervised machine learning techniques were used within QUARTZ; with a support vector machine used to create an image quality score^17^ and deep learning was used to develop an algorithm to distinguish between arterioles and venules.^18^ QUARTZ measures of width (µm^26^), total vessel area (mm^2^), tortuosity (arbitrary units)^15 27^ and variance of widths along a vessel segment, were averaged for each image (weighted by the length of each vessel segment), separately for arterioles and venules. Person level averages were obtained by averaging across right and left eyes.

### Statistical analysis

Statistical analyses were carried out using STATA software (V.16, StataCorp LP). Retinal vessel widths and area showed normal distributions, tortuosity required log-transformation and within-vessel-width-variance required inverse square-root transformation to normalise distributions. Models were developed in UKB for men and women separately throughout, and externally validated in EPIC-Norfolk. We hypothesised that retinal vessel characteristics in relation to circulatory mortality might be modified by age, smoking status, presence of CVD/diabetes and use of BP lowering medications. Hence, two-way interactions between RV and age, smoking status and self-reported use of BP medication, prevalent diabetes and CVD were first examined in mutually adjusted Cox proportional hazard^28^ models for circulatory mortality. Interaction terms with p values <0.2 were then included along with main effects in Cox regressions models using backward elimination (p value set to 0.1).

Bootstrapping with 100 replications was used for internal validation to adjust model performance measures for optimism, including Harrel’s C-statistic for discrimination, R^2^ statistic (representing a measure of explained variation)^29^ and calibration slope (where a slope of 1.0 is ideal).^30^ The original beta coefficients were adjusted for shrinkage by multiplying the beta-coefficients by the optimism-adjusted calibration slope (presented online supplemental table S3), applied to the EPIC-Norfolk cohort to estimate C-statistic, R^2^ and calibration slopes and baseline hazard. Model performance was graphically assessed from plots of the observed probability of event at 5 years by deciles of predicted risk at 5 years in UKB and by octiles in EPIC-Norfolk.

FRS for incident fatal and non-fatal stroke use age, systolic BP, treatment of hypertension, presence of diabetes and smoking status^21^ and for MI risk scores additionally include total and high-density lipoprotein (HDL) cholesterol levels,^22^ with separate risk equations in men and women; there risk scores were applied to UKB and EPIC-Norfolk cohorts and were recalibrated to the baseline survival function within each cohort according to the 5-year survival rates. Following FRS criteria, participants reporting use of cholesterol lowering medications, diabetes or missing data on total or HDL cholesterol were excluded from all MI analyses.^22^ FRS models were also extended to include RV. Alternative models for incident fatal and non-fatal stroke and MI using age, smoking status, medical history (self-reported history of heart attack, stroke or diabetes and use of BP lowering medications) and RV only were developed in UKB following the same approach as for circulatory mortality. A medical history of MI did not preclude inclusion in models for incident stroke events, and vice-versa.

Sensitivity analyses restricted model development and validation to white ethnicity. Using EPIC-Norfolk, external validation was extended to a broader spectrum of incident cerebrovascular disease (ICD-10 I60-69; ICD-9 430-438) and incident ischaemic heart disease (ICD-10 I20-I25; ICD-9 410-414). We followed Transparent Reporting of a multivariable prediction model for Individual Prognosis or Diagnosis guidelines for reporting of model development and validation.^31^

## Results

**Figure 1:**
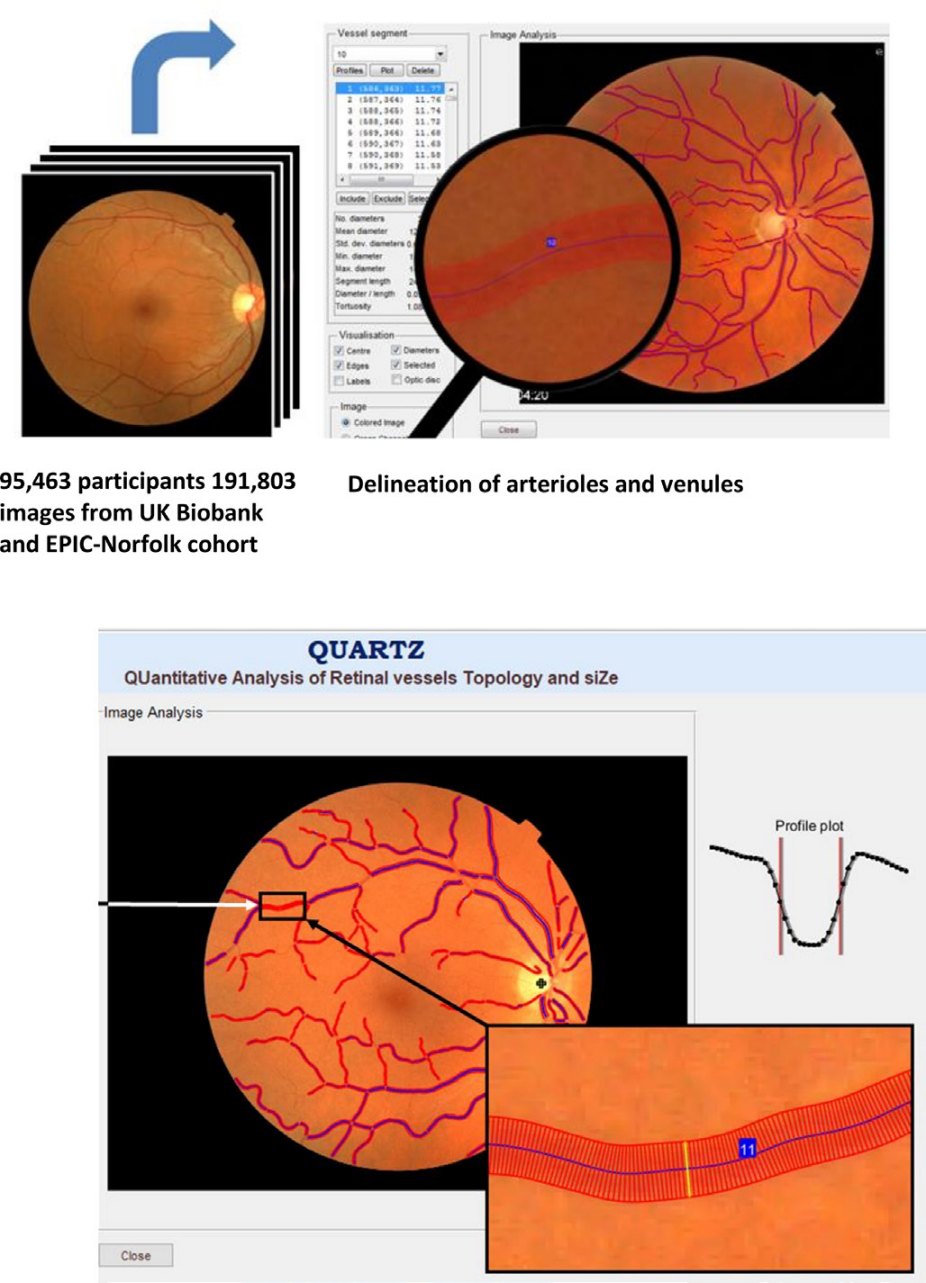
Fully automated retinal image processing of the vascular tree using artificial intelligence-enabled QUARTZ software.

Table 1 shows for UKB mean age at baseline was 56.8 years with median duration of follow-up 7.7 years after retinal image capture (maximum 8.2 years), and for EPIC-Norfolk, mean age was older (67.6 years) and median follow-up 9.1 years (maximum 12.4 years). Figure 1 is a visual representation of retinal image analysis using the QUARTZ software. Online supplemental figure S1 shows the number of UKB and EPIC-Norfolk participants and events available for circulatory mortality, incident stroke and incident MI analyses.

**Table 1:** Clinical characteristics at baseline eye assessment in UK Biobank (2009–2013) and from the third health check phase for EPIC-Norfolk (2004–2011)

Baseline characteristic	Mean (SD) or (%)	
	UK Biobank N=66 326	EPIC N=5955
Median duration of follow-up (years)	7.7	9.1
Age (years)	56.8 (8.2)	67.6 (7.6)
Female (%)	55.0%	57.1%
Ethnicity (%)		
White	92.0%	99.5%
Black	2.5%	0.1%
Asian	2.5%	0.0%
Other	2.5%	0.2%
Unknown/did not answer	0.6%	0.0%
Smoking (%)		
Never smoker	56.7%	49.7%
Occasionally	2.6%	N/A
Ex-smoker	34.0%	44.2%
Current smoker	6.1%	4.5%
Prefer not to say/missing	0.5%	1.6%
BMI (kg/m²)	27.2 (4.7)	26.8 (4.3)
Systolic BP (mm Hg)	136.8 (18.3)	135.7 (16.6)
Diastolic BP (mm Hg)	81.5 (10.0)	78.5 (9.3)
Total cholesterol (mmol/L)	5.7 (1.1)	5.4 (1.1)
LDL cholesterol (mmol/L)	3.5 (0.9)	3.2 (1.0)
HDL cholesterol (mmol/L)	1.5 (0.4)	1.5 (0.4)
Triglycerides (mmol/L)	1.7 (1.0)	1.7 (0.9)

	UK Biobank	EPIC
Image quality*	0.9 (0.1)	0.9 (0.1)
Arteriolar width (µm)	86.9 (7.9)	75.1 (6.4)
Venular width (µm)	103.1 (13.0)	91.4 (10.8)
Arteriolar tortuosity†	4.4 (1.6)	4.3 (1.6)
Venular tortuosity†	3.1 (1.4)	3.2 (1.3)
Arteriolar vessel area (mm²)	1.8 (0.8)	2.0 (0.7)
Venular vessel area (mm²)	2.5 (0.9)	2.6 (0.7)
Arteriolar segment SD^−1^ (µm^−1^)	0.08 (0.03)	0.13 (0.03)
Venular segment SD^−1^ (µm^−1^)	0.10 (0.03)	0.14 (0.04)
Use of medications		
Blood pressure lowering	19.6%	35.1%
Cholesterol lowering	17.9%	22.3%
Prefer not to report/missing	1.2%	N/A
Self-reported history		
Heart attack (%)	1.9%	3.1%
Stroke (%)	1.4%	2.0%
Prefer not to report/missing	1.2%	N/A
Diabetes (%)	4.9%	4.0%

Values are mean (SD) or (%).

Missing data were BMI n=287, blood pressure n=207 in UK Biobank only. Framingham risk score-based models for incident MI that used lipids missing data were as follows after excluding those with prevalent events (MI or diabetes) or using lipid lowering therapy, n= 6805 for total cholesterol or HDL cholesterol for UK Biobank. Other missing variables for UK Biobank were LDL cholesterol n=4628; triglycerides n=4576; and for EPIC-Norfolk—total cholesterol n=428, LDL cholesterol n=510, HDL cholesterol n=427, triglycerides n=428)

*Image quality score generated by QUARTZ, values range from 0.6 to 1.0, higher values indicate higher image quality.

†*Geometric mean exponentiated SD of the log-transformed values; the 95% range for the geometric mean is from (geometric mean÷GSD^2^) to (geometric mean×GSD^2^).*

BMI, body mass index; BP, blood pressure; EPIC, European Prospective Investigation into Cancer; HDL, high-density lipoprotein; LDL, low-density lipoprotein; QUARTZ, QUantitative Analysis of Retinal vessels Topology and siZe.

### Circulatory mortality

**Figure 2:**
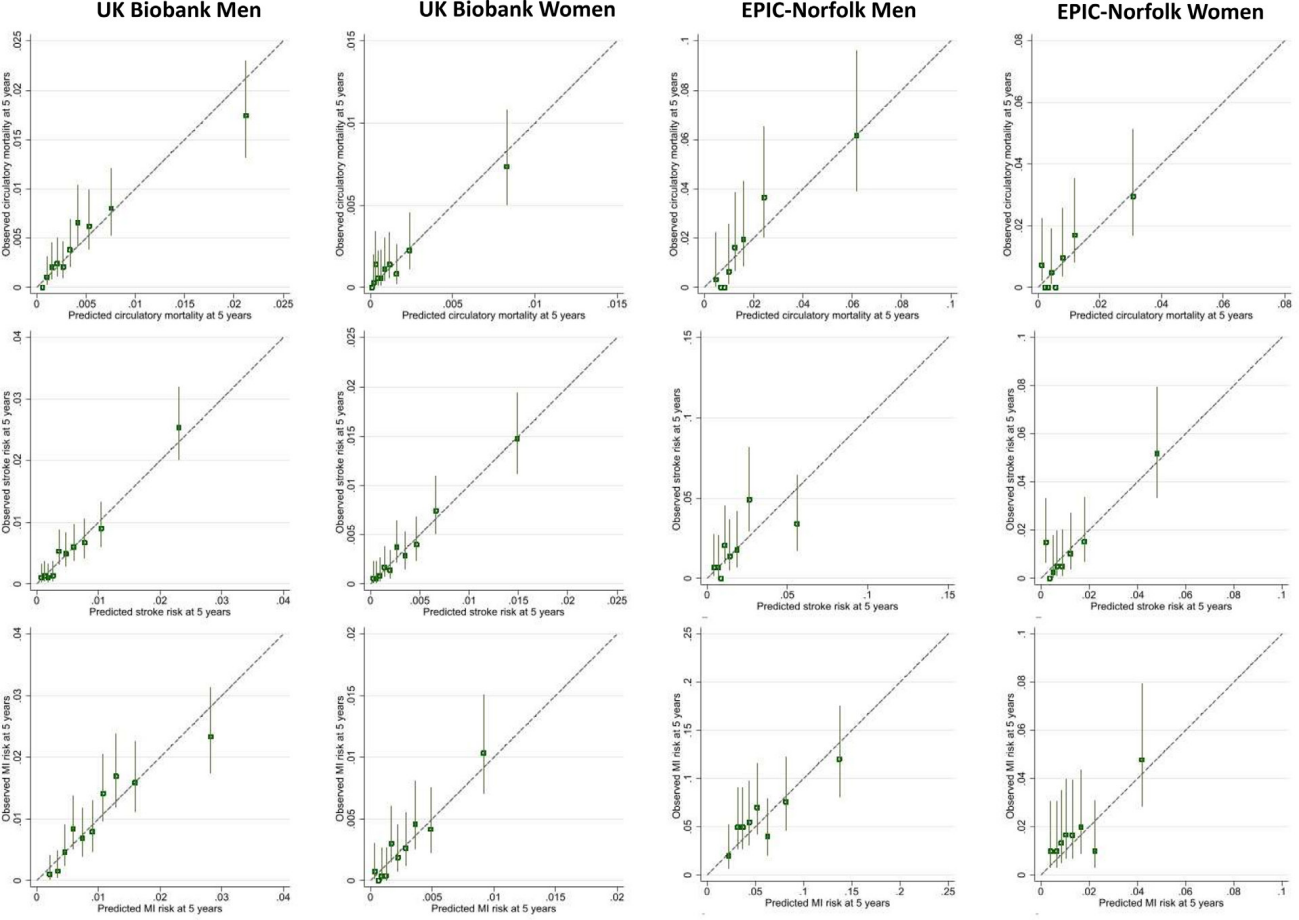
Observed risk of outcome at 5 years by deciles of predicted risk in Biobank and eights of predicted risk in EPIC-Norfolk. Predicted risk based on model using age, smoking, medical history and retinal vasculomatry. EPIC, European Prospective Investigation into Cancer. Vertical lines around symbols are the 95% confidence intervals. Dotted lines represent perfect calibration. The scale of the vertical and horizontal axes represent the probability e.g., 0.1 equates to a 10% risk of event by 5 years.

64 144 UKB participants with 327 circulatory deaths and 5862 EPIC-Norfolk participants with 201 circulatory deaths were included. In men, arteriolar and venular width, tortuosity and width-variance were identified as statistically significant predictors of circulatory mortality. In women, arteriolar and venular area and width, venular tortuosity and venular width-variation contributed to risk prediction. RV effects on circulatory mortality were modified by smoking status, BP medications and history of MI. In men and women, optimism adjusted C-statistics (0.75–0.77) and R^2^ (0.33–0.44) statistics in UKB and EPIC-Norfolk, were reasonably high (table 2, online supplemental table S1 for full model diagnostics; online supplemental tables S2 and S3 for regression coefficients). In UKB men, predicted risks were closely aligned with observed risks. A similar picture emerged for EPIC-Norfolk men cohort, with about double the risk of circulatory mortality, half the numbers of events and being about a decade older at retinal image capture (figure 2). UKB women showed a wide separation of risk groups and close alignment of predicted and observed risks even, at low risks (<0.5%). Calibration plots for EPIC-Norfolk women were less clear due to the lower number of events available, hence 95% CI around predictions were wider (figure 2).

**Table 2:** Optimism adjusted model performance (95% CIs) for prediction of circulatory mortality, incident stroke and myocardial infarction models developed in UK Biobank cohort (2009–2018) with external validation in EPIC-Norfolk cohort (2004–2018)

Model	UK Biobank men	UK Biobank women	EPIC-Norfolk men	EPIC-Norfolk women
	Circulatory mortality (number of events/sample size)			
**Age, smoking, medical history+RV**	(227/29 257)	(100/35 887)	(114/2516)	(87/3346)
Calibration slope	0.913 (0.800 to 1.026)	0.857 (0.732 to 0.982)	1.084 (0.888 to 1.279)	0.872 (0.674 to 1.070)
C-statistic	0.749 (0.720 to 0.779)	0.763 (0.717 to 0.810)	0.774 (0.732 to 0.815)	0.748 (0.692 to 0.805)
R^2^	0.369 (0.310 to 0.427)	0.443 (0.369 to 0.518)	0.392 (0.302 to 0.482)	0.333 (0.228 to 0.438)
	Stroke (number of events/sample size)			
**FRS for stroke**	(245/28 573)	(201/35 266)	(98/2432)	(113/3276)
Calibration slope	0.908 (0.769 to 1.047)	0.919 (0.764 to 1.074)	0.819 (0.552 to 1.087)	0.943 (0.734 to 1.152)
C-statistic	0.736 (0.706 to 0.766)	0.736 (0.702 to 0.770)	0.682 (0.629 to 0.735)	0.732 (0.682 to 0.781)
R^2^	0.295 (0.233 to 0.358)	0.310 (0.240 to 0.379)	0.199 (0.098 to 0.300)	0.309 (0.215 to 0.402)
**Age, smoking, medical history+RV**				
Calibration slope	0.896 (0.767 to 1.025)	0.860 (0.729 to 0.991)	0.808 (0.571 to 1.045)	0.780 (0.603 to 0.958)
C-statistic	0.729 (0.699 to 0.759)	0.753 (0.721 to 0.784)	0.691 (0.637 to 0.746)	0.714 (0.660 to 0.768)
R^2^	0.315 (0.256 to 0.375)	0.352 (0.289 to 0.416)	0.213 (0.113 to 0.314)	0.274 (0.179 to 0.369)
	Myocardial infarction (number of events/sample size)			
**FRS for confirmed MI**	(275/19 150)	(118/26 584)	(166/1622)	(99/2440)
Calibration slope	1.216 (0.994 to 1.439)	1.036 (0.813 to 1.260)	1.567 (1.210 to 1.924)	0.834 (0.583 to 1.085)
C-statistic	0.706 (0.678 to 0.734)	0.758 (0.718 to 0.798)	0.689 (0.650 to 0.728)	0.688 (0.640 to 0.737)
R^2^	0.235 (0.175 to 0.295)	0.345 (0.256 to 0.433)	0.233 (0.153 to 0.312)	0.208 (0.109 to 0.308)
**Age, smoking, medical history+RV**				
Calibration slope	0.836 (0.673 to 0.999)	0.803 (0.590 to 1.016)	0.905 (0.655 to 1.156)	0.786 (0.517 to 1.054)
C-statistic	0.675 (0.647 to 0.703)	0.709 (0.669 to 0.749)	0.641 (0.598 to 0.683)	0.650 (0.593 to 0.707)
R^2^	0.178 (0.118 to 0.238)	0.226 (0.136 to 0.316)	0.150 (0.077 to 0.224)	0.162 (0.067 to 0.256)

Framingham risk scores (FRS) for incident stroke and myocardial infarction are also presented.

Estimates for calibration slope, C-statistic and R^2^ values are given with bootstrapped 95% CI in parenthesis.

FRS, Framingham risk score; RV, retinal vasculometry.

### Incident stroke

63 839 UKB participants with 446 incident strokes and 5708 EPIC-Norfolk participants with 211 incident stroke events after retinal image capture were included (online supplemental figure S1). In UK-Biobank, FRS C-statistic was 0.74 in men and 0.74 in women (table 2) with lower values in EPIC-Norfolk; approximately one-third of the variation in stroke-risk incidence was explained by R^2^ (less so in EPIC-Norfolk men). Observed risks were more aligned with predicted risks in men than in women (online supplemental figure S2). Addition of RV to FRS did not improve model performance statistics overall (online supplemental table S4, figure S2).

Models based on age, smoking status, medical history and RV showed similar performance to FRS with C-statistic of 0.73 in men and 0.75 in women and marginally improved R^2^ values in UKB (table 2; full model diagnostics online supplemental table S4). As for FRS, performance metrics were lower in EPIC-Norfolk. Multivariable models (online supplemental tables S2 and S3) showed venular and arteriolar tortuosity and width were predictors of stroke in men and women and additionally venular/arteriolar area in women with some modification by smoking status, BP medications and history of MI. Calibration plots showed risk predictions closer to the 45° line particularly at lower levels of predicted risk in women (online supplemental figure S2).

### Incident MI

45 734 UKB participants with 393 incident MI and 4062 in EPIC-Norfolk with 265 incident MI after retinal image capture were included (online supplemental figure S1). In UKB, FRS C-statistics were 0.71 in men and 0.76 in women with approximately one-quarter (24%) of the variation in MI risk explained by FRS in men and 35% in women (table 2). In EPIC-Norfolk, with approximately 5× the risk of MI, performance statistics were lower. Calibration plots for FRS showed better alignment of observed and predicted risks in men compared with women (online supplemental figure S3). Addition of RV to FRS did not improve model performance overall (online supplemental table S5, figure S3). Compared with FRS alone a simpler model based on age, smoking status, medical history and RV performed marginally less well in men and women in both cohorts (online supplemental tables S2 and S5, figure S3). Multivariable models for MI using RV (online supplemental tables S2 and S3) showed arteriolar and venular width, venular width variability and arteriolar area were predictors in men, whereas for women venular tortuosity, venular/arteriolar area and venular width variability were predictors. RV effects were modified by smoking status.

### Cases in top quintile of risk scores

For circulatory mortality models based on age, smoking status medical history and RV captured between 52% and 65% of cases of circulatory mortality in the top quintile of the risk score distribution (table 3). For incident stroke, RV based models compared with FRS captured about 5% more cases in UKB men and 8% more cases in UKB women and 3% more EPIC-Norfolk men in the top quintile of risk scores (table 3) but 1.8% fewer EPIC-Norfolk women. However, for MI, FRS captured more cases of MI in the top quintile of risk. Considering stroke and MI scores combined, the simpler RV models captured more cases in the top quintile than FRS for UKB men and women, and similar proportions in EPIC-Norfolk men and women.

**Table 3:** Percentage of circulatory mortality, incident stroke and incident MI events (after retinal image capture) in top quintile of risk score distributions for UK Biobank and EPIC-Norfolk

Model	UK Biobank		UK Biobank		EPIC-Norfolk		EPIC-Norfolk	
	Men		Women		Men		Women	
	Number, % of all circulatory mortality in top quintile of circulatory mortality risk score distribution							
Age, smoking, medical history+RV	126	55.5%	65	65.0%	63	55.3%	45	51.7%
	**Number, % of all incident stroke in top quintile of stroke risk score distribution**							
FRS stroke	115	46.9%	100	49.8%	37	37.8%	55	48.7%
Age, smoking, medical history+RV	133	54.3%	114	56.7%	40	40.8%	53	46.9%
	**Number, % of all incident MI in top quintile of MI risk score distribution**							
FRS confirmed MI	116	42.2%	59	50.0%	68	41.0%	37	37.4%
Age, smoking, medical history+RV	109	39.6%	58	49.2%	65	39.2%	33	33.3%
	**Number, % of all incident stroke or MI in top quintile of stroke or MI risk score distribution**							
FRS confirmed MI or FRS stroke	259	49.8%	181	56.7%	120	45.5%	108	50.9%
Age, smoking, medical history+RV	264	50.8%	190	59.6%	119	45.5%	104	49.9%

FRS, Framingham risk scores; MI, myocardial infarction; RV, retinal vasculometry.

### Sensitivity analyses

Restricting model development and validation to those of white ethnicity did not materially alter model performance for any of the models presented. FRS and all RV models for stroke showed systematically improved external validation for outcomes based on inclusion of all incident cerebrovascular disease in EPIC-Norfolk (online supplemental table S4) far right-hand column and (online supplemental figure S4), especially in women. In contrast, for *all* incident ischaemic heart disease in EPIC-Norfolk, performance of FRS and RV models remained remarkably unchanged in men but marginally improved in women (online supplemental table S5 far right-hand column and online supplemental figure S5).

## Discussion

This study compares risk predictions using AI-enabled RV with established CVD risk-algorithms. To the best of our knowledge it represents the largest population-based study of RV. Importantly, external validation of the prediction models was carried out in a separate large cohort, which is uncommon in this field. Our automated AI-enabled system extracts the retinal vascular tree over the entire retinal image (figure 1), distinguishes between arterioles and venules and provides measures of tortuosity, width-variance and area, in addition to vessel width. Risk models showed that all RV components contributed to risk prediction. Adding RV to FRS resulted in marginal changes in the prediction of stroke or MI. However, a simpler non-invasive risk score based on age, sex, smoking status, medical history and RV yielded comparable performance to FRS, without the need for blood sampling or BP measurement. Prediction of circulatory mortality using age, sex, smoking status, medical history and RV has not been reported previously, and yielded the highest model performance in terms of C-statistics R^2^ statistics and agreement between observed and predicted risks, even at lower levels of risk, in both the internal and external validation cohorts.

### Comparisons with other studies

Prospective associations have been largely based on retinal vessel width with mortality,^12^ incident stroke^12^ and with CHD (in women, not men),^10 32^ from restricted measurement areas of the retina.^10 33^ Measurements are often not automated, requiring operator involvement, which limits application to large populations. In agreement with others, our models show that both arteriolar and venular vasculometry contribute to risk prediction,^10 34^ and this aligns with our previous work.^15 27 35^ Seidelmann *et al* reported that narrower central retinal artery and wider central retinal vein equivalent dimensions offered significant additional information to equations for incident atherosclerotic CVD risk,^10^ especially in women, but C-statistics were modest (between 0.55 and 0.57) compared with the much higher levels in the current study (ie, between 0.70 and 0.77). Our RV models generally performed better in women and may indicate that microvascular dysfunction contributes more to CHD pathogenesis in women than in men, as they have smaller coronary arteries exhibiting more diffuse ‘non-obstructive’ atherosclerosis,^36^ with a larger burden of coronary microvascular disease,^37^ leading to higher morbidity and mortality.^38^ A recent study using the UKB data source in fewer participants (54 813 vs 65 144 in this study), showed that retinal vessel density and fractal dimensions (extracted from the entire image after deep learning vessel segmentation without distinction between arterioles and venules) were associated with other health outcomes, including overall mortality, hypertension and congestive heart failure, but did not report on risk prediction performance.^39^ Moreover, there was no consistent evidence of associations with incident circulatory disease, and cerebrovascular disease and associations with incident MI were null.^39^ Another study in a sub-set of UKB participants (n=5663) with both retinal and cardiovascular MRI used deep learning/AI approaches to estimate structural cardiac indices as intermediaries for predicting MI.^40^ However, given their approach, specific retinal features of importance remain unclear.

European SCORE CVD risk score,^8^ QRISK3 risk score^41^ and the American College of Cardiology/American Heart Association CVD risk score algorithms have already been evaluated in UKB. The published C-statistics for these three risk scores were 0.77, 0.74 and 0.74, respectively, with 95% CI that overlap with values for the simpler RV model presented in this study. However, the novel C-statistics for circulatory mortality reported in this study are higher. Our approach of focussing on the retinal microvasculature as a key prognostic marker of incident cardiovascular outcomes and circulatory mortality is supported by saliency maps presented in a study using end-to-end AI of retinal images to estimate the extent of coronary artery calcium scores in cross-sectional associations,^42^ with C-statistics for incident CVD varying between 0.68 and 0.76. Our model using RV together with easily attainable data including age, smoking status, sex and a brief medical history, is simple, non-invasive and exhibits performance that is comparable, or even better than, current risk algorithms, including end-to-end AI approaches.

Online supplemental tables S2 and S3 present the regression-coefficients for the RV models for circulatory mortality, incident stroke and incident MI. Beta-coefficients with p values ≤0.1 (as defined by our backward stepwise elimination for model development) are retained in the risk prediction equation. Regression coefficients with p>0.1 were therefore not included in the model. It is usual to present both main effects and interaction effects in the same model even if the main effect is not statistically significant. However, in risk prediction only coefficients that contribute to risk discrimination are retained and coefficients that are not formally statistically significant, as defined a priori, will not add to discrimination, and are therefore not included in the final risk equation. This may at first seem counterintuitive, but it is evident that certain RV features are important in risk prediction because they are related to (or potentially affected by) other factors such as smoking, presence of CVD and BP lowering medications, which is biologically plausible and supported by other evidence.^12 14 15 43–48^

### Strengths and limitations

Model development in UKB provided a large sample size and number of prospective events. QUARTZ successfully processed a high percentage (77%) of retinal images captured by non-experts providing ‘vasculomic’ indices of vascular health. External validation in an older higher risk cohort (EPIC-Norfolk) replicated the findings, and models were also robust to inclusion of a wider spectrum of cerebrovascular and ischaemic heart disease events.

UKB and EPIC-Norfolk are ‘healthy’ cohorts with relatively low event rates compared with other geographically similar middle-aged cohorts.^49^ Prevalence of current smoking was very low in UKB (6%) and limited the ability to examine interactions with RV. Although we did not find limiting the analysis to those of white ethnicity materially altered the results, the proportion of non-white participants in UKB is low. RV may relate to microvascular endothelial function elsewhere in the body and may underpin the causal pathways behind prognostic models, which may differ with ethnicity. Confirmation of model performance in other cohorts with higher CVD rates and in different (especially non-white) ethnic groups would be informative.

### Implications and conclusions

Retinal imaging is established within clinic and hospital eye care and in optometric practices in the US and UK. AI-enabled vasculometry risk prediction is fully automated, low cost, non-invasive and has the potential for reaching a higher proportion of the population in the community because of ‘high street’ availability and because blood sampling or sphygmomanometry are not needed. RV is a microvascular marker, hence offers better prediction for circulatory mortality and stroke compared with MI which is more macrovascular, except perhaps in women. In the general population it could be used as a non-contact form of systemic vascular health check, to triage those at medium-high risk of circulatory mortality for further clinical risk assessment and appropriate intervention. In 2017–2018 in the UK, 41% of 40–74 years old attended their primary care NHS Health Check, which includes QRISK based screening for CVD.^50^ With a trend towards lower attendance in more recent years (ie, from 2012 onwards), and socioeconomic inequalities in attendance (where younger ages, males, those more deprived and certain ethnic groups were less likely to attend),^50^ this ‘high street’ RV approach could directly feed into primary medical services and help achieve greater screening coverage (under the assumption that this age group are likely to attend optometric practice for visual correction, especially with the onset of presbyopia). In addition, this would offer a novel approach to identify those at high risk of circulatory mortality, which are not currently screened for. While a high percentage of retinal images in this study captured by non-expert personnel were of sufficient quality to be used for RV quantification (~80%), we would expect this to be improved with fundus imaging carried out by healthcare practitioners, such as those working in optometric practice. However, moving forward experimental evidence would be needed to formally assess the effectiveness on CVD prevention before advocating implementation. Despite this, having a further low cost, accessible, non-invasive screening test in the community to encourage clinical risk assessment uptake in the community (in addition to current screening approaches), is highly likely to help prolong disease-free status in an ever-ageing population with increasing comorbidities, and assist with minimising healthcare costs associated with lifelong vascular diseases.

## Data Availability

Data may be obtained from a third party and are not publicly available. The data supporting the results reported here are available through the UK Biobank https://www.ukbiobank.ac.uk/enable-your-research/apply-for-access.
